# A novel chiral stationary phase LC-MS/MS method to evaluate oxidation mechanisms of edible oils

**DOI:** 10.1038/s41598-017-10536-2

**Published:** 2017-08-30

**Authors:** Junya Ito, Naoki Shimizu, Eri Kobayashi, Yasuhiko Hanzawa, Yurika Otoki, Shunji Kato, Takafumi Hirokawa, Shigefumi Kuwahara, Teruo Miyazawa, Kiyotaka Nakagawa

**Affiliations:** 1 Food and Biodynamic Chemistry Laboratory, Graduate School of Agricultural Science, Tohoku University, Sendai, 980-0845 Japan; 2 Department of Cell Biology, Division of Host Defense Mechanism, Tokai University School of Medicine, Isehara, Kanagawa 259-1193 Japan; 3 Laboratory of Applied Bioorganic Chemistry, Graduate School of Agricultural Science, Tohoku University, Sendai, 980-0845 Japan; 4 New Industry Creation Hatchery Center (NICHe), Tohoku University, Sendai, 980-8579 Japan

## Abstract

The elucidation of lipid oxidation mechanisms of food is vital. In certain lipids, characteristic lipid hydroperoxide isomers are formed by different oxidation mechanisms (*i.e*., photo-oxidation or auto-oxidation). For example, linoleic acid is photo-oxidized to 13-9*Z*, 11*E*-hydroperoxyoctadecadienoic acid (HPODE), 12-9*Z*,13*E*-HPODE, 10-8*E*,12*Z*-HPODE and 9-10*E*,12*Z*-HPODE, whereas 13-9*Z*, 11*E*-HPODE, 13-9*E*,11*E*-HPODE, 9-10*E*,12*Z*-HPODE and 9-10*E*,12*E*-HPODE are formed by auto-oxidation. Therefore, we considered that oxidation mechanisms could be evaluated by analyzing these characteristic positional and *cis/trans* lipid hydroperoxide isomers. In this study, we developed a novel chiral stationary phase LC-MS/MS (CSP-LC-MS/MS) method to analyze the positional and *cis/trans* isomers of HPODE, with the use of a chiral column and sodium ion. Also, as an application of the method, either light-exposed or heated edible oils were treated with lipase to hydrolyze triacylglycerols. The resultant fatty acids including HPODE isomers were analyzed with the developed method. As a result, HPODE isomers characteristic to photo-oxidation were certainly detected in light-exposed edible oils. On the other hand, in heated edible oils, the HPODE isomers characteristic to auto-oxidation were largely increased. Thus, the combination of the developed CSP-LC-MS/MS method with lipase proves to be a powerful tool to evaluate the involvement and mechanisms of lipid oxidation in the process of food deterioration.

## Introduction

Lipid oxidation is one of the main causes of food deterioration in terms of flavor, taste and nutritional value^1–3^. Serious biological effects caused by the intake of oxidized lipids are also reported in some animal studies^4, 5^. Therefore, the elucidation of lipid oxidation mechanisms of food (*i.e.*, edible oils) is vital. The level of lipid oxidation in edible oil is typically evaluated by measuring its peroxide value (POV)^6, 7^. POV is determined by measuring the amount of iodine that forms as a result of a reaction between peroxides and iodide ions. Thereby, POV can only assess the concentration of lipid hydroperoxides and lipid oxidation mechanisms cannot be evaluated. If the oxidation mechanisms that happen in edible oils can be identified, effective preventive measures specific to the oxidation mechanism can be developed.

**Figure 1:**
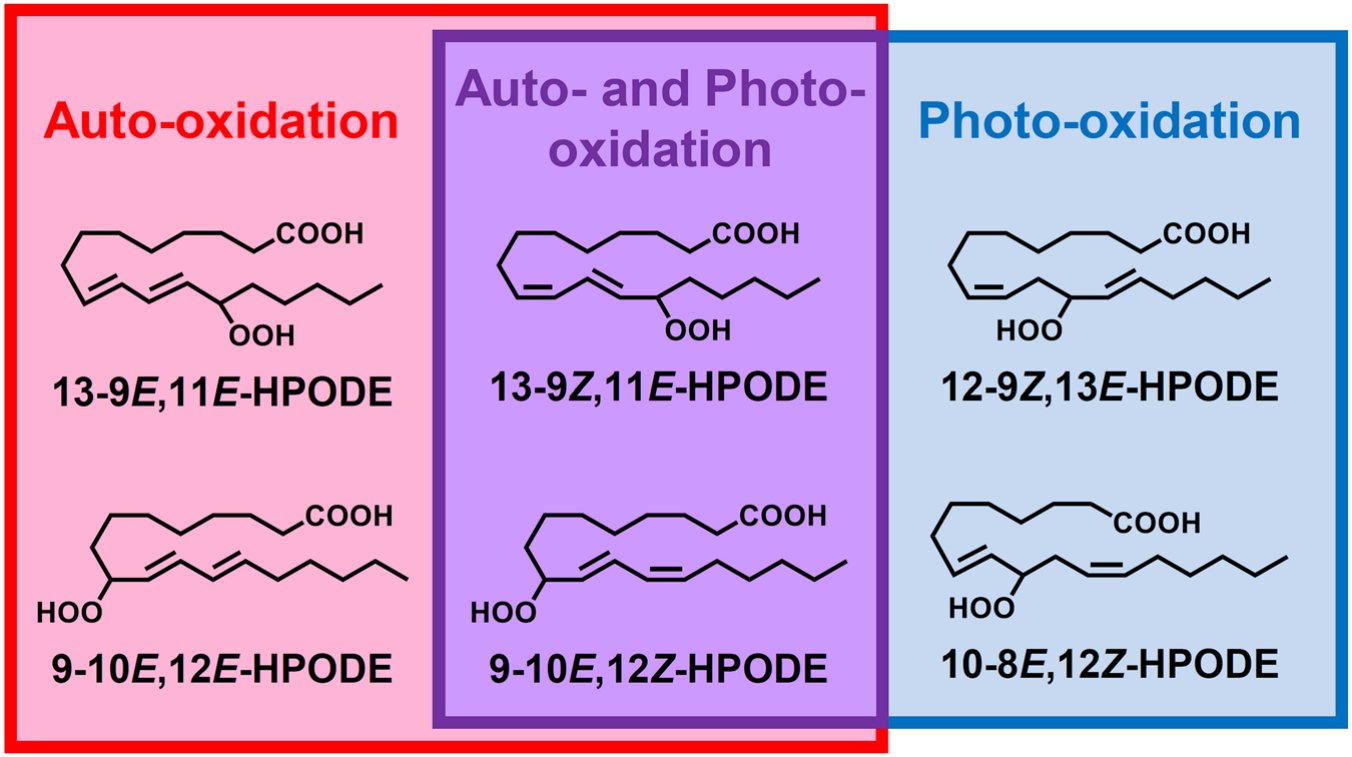
Chemical structures of LA and six HPODE isomers (13-9*Z*,11*E*-HPODE, 13-9*E*,11*E*-HPODE, 12-9*Z*,13*E*-HPODE, 10-8*E*,12*Z*-HPODE, 9-10*E*,12*Z*-HPODE and 9-10*E*,12*E*-HPODE).

Lipid hydroperoxides, the primary product of lipid oxidation, form through different oxidation mechanisms: photo-oxidation (singlet oxygen induced oxidation), auto-oxidation (radical oxidation) and/or enzymatic oxidation (lipoxygenase induced oxidation)^8–11^. In certain lipids, each oxidation mechanism yields characteristic lipid hydroperoxide isomers. For example, linoleic acid (LA) is photo-oxidized to 13-9*Z*,11*E*-hydroperoxyoctadecadienoic acid (HPODE), 12-9*Z*,13*E*-HPODE, 10-8*E*,12*Z*-HPODE and 9-10*E*,12*Z*-HPODE^12, 13^, whereas 13-9*Z*,11*E*-HPODE, 13-9*E*,11*E*-HPODE, 9-10*E*,12*Z*-HPODE and 9-10*E*,12*E*-HPODE are formed by auto-oxidation^14, 15^ (Fig. 1). Therefore, we considered that the oxidation mechanisms of lipids can be identified by analyzing the characteristic positional and *cis/trans* isomers of lipid hydroperoxides. Such information regarding the oxidation mechanism of lipids will be valuable in efforts for developing preventive methods against lipid oxidation in foods.

Among lipids, fatty acids are fundamental molecules that constitute various other lipids (*e.g*., triacylglycerols, cholesterol esters and phospholipids). Especially, LA is a fatty acid that constitutes many food lipids (*i.e*., edible oils); thus, the LA hydroperoxide isomers (*i.e*., HPODE isomers) could reflect the oxidation mechanisms of lipids in food.

In previous studies, HPODE isomers were reduced or derivatized to produce hydroxyl fatty acid (*i.e*., HODE (hydroxy octadecadienoic acid)) isomers or their derivatives^16, 17^. And these HODE and their derivatives isomers were separated and analyzed. However, these previous methods were not able to discriminate the HODE that was produced from HPODE via reduction and the HODE that originally existed in the sample. In addition, since HPODE is a primary oxidation product and HODE is a secondary oxidation product, the measurement of HPODE is considered to be important in order to evaluate the initial lipid oxidation in food. However, analysis of the positional and *cis/trans* isomers of HPODE is difficult even with the use of mass spectrometry and nuclear magnetic resonance (NMR).

To solve the matter, we have recently developed a method to identify the positional isomers of HPODE (*e.g*., 13-HPODE, 12-HPODE, 10-HPODE and 9-HPODE) by performing MS/MS analysis in the presence of alkali metals (*e.g*., sodium ions)^18^. Although the use of alkali metals enable highly sensitive and selective analysis of HPODE isomers, because the HPODE *cis/trans* isomers (*e.g*., 9-10*E*,12*Z*-HPODE and 9-10*E*,12*E*-HPODE) are detected by the same selected reaction monitoring (SRM) pairs^18^, MS/MS *per se* was not able to separate the HPODE *cis/trans* isomers in our previous studies. Therefore, in this study, we applied a combination of MS/MS and chiral stationary phase LC (CSP-LC-MS/MS), and achieved the simultaneous separation of positional and *cis/trans* isomers of HPODE for the first time. Also, as an application of the method, three types of edible oils were either light-exposed or heated. The oxidized edible oils were subsequently treated with lipase to hydrolyze triacylglycerols. The resultant fatty acids including HPODE isomers were analyzed with the developed method. Based on the results, it was suggested that the present method enables the identification of oxidation mechanisms in edible oils. Hence, the herein developed CSP-LC-MS/MS method appears to be a powerful and novel tool to evaluate lipid oxidation mechanisms in foods.

## Results and Discussion

### Preparation of HPODE isomer standards and development of the CSP-LC-MS/MS method

HPODE isomer standards were prepared by the photo-oxidation of LA according to our previous study^18^. The prepared standards (*i.e*., 13-9*Z*,11*E*-HPODE, 13-9*E*,11*E*-HPODE, 12-9*Z*,13*E*-HPODE, 10-8*E*,12*Z*-HPODE, 9-10*E*,12*Z*-HPODE and 9-10*E*,12*E*-HPODE) were each highly pure (>95%) judged from the data of^1^H NMR and infusion MS spectra (data not shown). As reported in previous studies, fatty acid hydroperoxides (*i.e*., HPODE) can be used to prepare various glycerolipid hydroperoxides (*e.g*., phospholipid hydroperoxides)^10, 11, 19, 20^. Accordingly, the synthetic method and the prepared HPODE standards could also be beneficial for studies involving other lipid hydroperoxides (*e.g*., metabolome analysis and cell culture assays).

**Figure 2:**
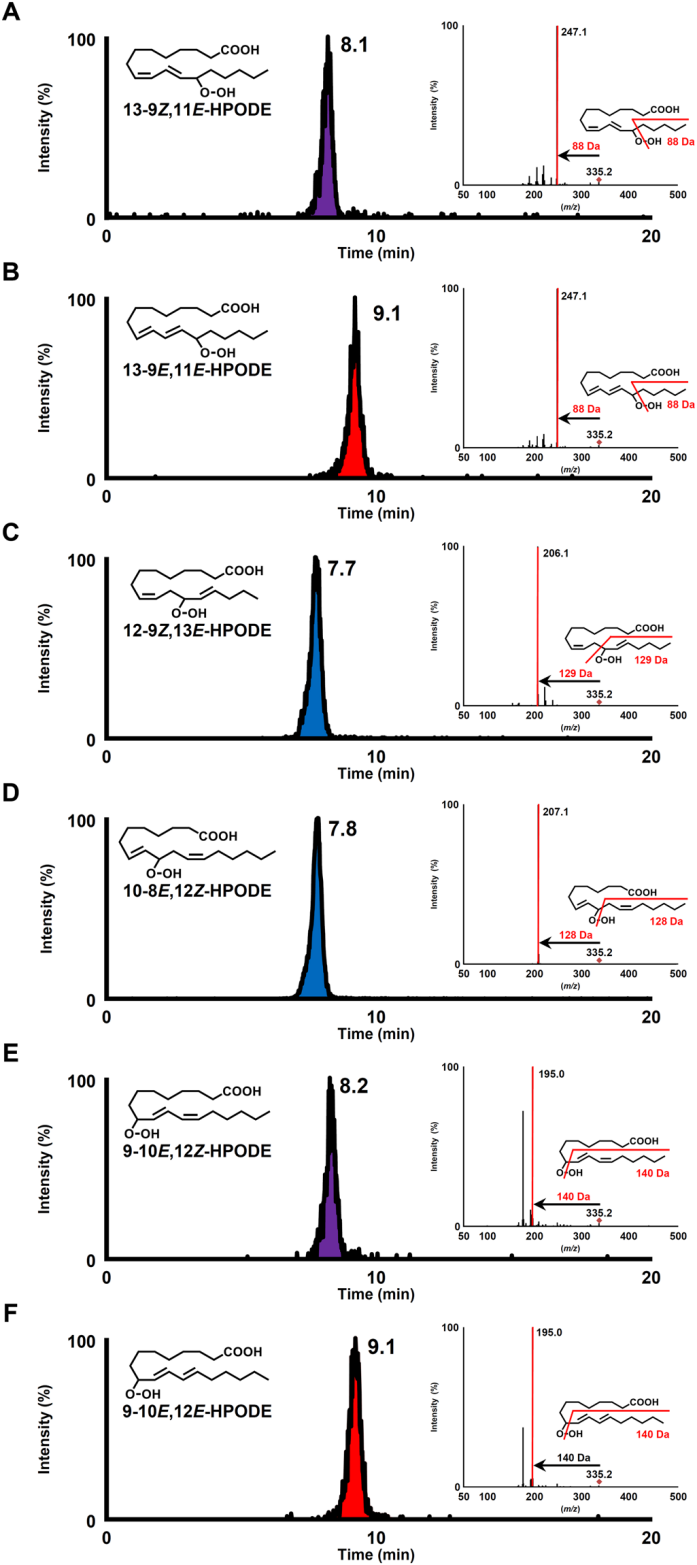
CSP-LC-MS/MS chromatogram and product ion mass spectra (MS/MS) of standard HPODE isomers. HPODE isomers were determined using the SRM mode as follows: 13-9*Z*,11*E*-HPODE and 13-9*E*,11*E*-HPODE, *m/z* 335.2 > 247.1 (**A**,**B**); 12-9*Z*,13*E*-HPODE, *m/z* 335.2 > 206.1 (**C**); 10-8*E*,12*Z*-HPODE, *m/z* 335.2 > 207.1 (**D**); 9-10*E*,12*Z*-HPODE and 9-10*E*,12*E*-HPODE, *m/z* 335.2 > 195.1 (**E**,**F**). Detailed analytical conditions are described in the Materials and Method section.

CSP-LC-MS/MS analysis of the prepared isomer standards was conducted using a chiral column (*i.e*., CHIRALPAK IB N-3) and a micrOTOF-Q II mass spectrometer. In this method, sodium ions are added as a post column reagent to enable the selective MS/MS analysis of fatty acid hydroperoxides as we reported previously^10^. By adding sodium, fragmentations patterns similar to previous studies^18^ were obtained during CSP-LC-MS/MS analysis (Fig. 2). The results indicate that the use of sodium ions allow for the highly sensitive and selective analysis of fatty acid hydroperoxides. One reason why the addition of sodium ions during MS/MS analysis of HPODE enables unique fragmentations may be its contribution to α-cleavage and Hock cleavage. α-Cleavage is a type of fragmentation mechanism observed during MS/MS analysis^21^, and Hock cleavage is a decomposition reaction that forms aldehydes in the catalysis of Lewis acids or protons^22^. In this study, fragment ions used for SRM pairs were consistent with the decomposition products that were estimated to be generated by α-cleavage (*i.e*., 13-9*Z*,11*E*-HPODE, 13-9*E*,11*E*-HPODE and 10-8*E*,12*Z*-HPODE) or Hock cleavage (*i.e*., 9-10*E*,12*Z*-HPODE and 9-10*E*,12*E*-HPODE) (Supplementary Information 1). Based on these results, it can be assumed that α-cleavage, Hock cleavage, or a similar reaction occurrs during the collision induced dissociation in CSP-LC-MS/MS analysis, suggesting that sodium ions contribute to the catalysis of these reactions and/or the stabilization of reaction products (fragment ions). To support this hypothesis, in a previous study, Porter *et al*. reported that Hock cleavage occurs during the MS analysis of cholesterol ester hydroperoxide isomers in the existence of silver ions^23^. Whereas Porter *et al*. used silver ions, our method uses a low concentration sodium ion (alkali metal ion), and thereby reduces the burden on the MS system.

While MS/MS analysis can clearly separate the HPODE positional isomers, the separation of the HPODE *cis/trans* isomers (*e.g*., 9-10*E*, 12*Z*-HPODE and 9-10*E*,12*E*-HPODE) cannot be achieved mass-spectrometrically, because the *cis/trans* isomers are detected by the same SRM pairs. Thus, HPODE *cis/trans* isomers need to be separated chromatographically using LC. In previous studies, HPODE *cis/trans* isomers and their derivatives have been separated by straight phase LC methods^24, 25^. However, because the mobile phase is limited to solvents like hexane, highly sensitive analysis with MS/MS is difficult with straight phase LC. Hence, we employed reversed phase-CSP-LC-MS/MS method which allows for the highly sensitive MS/MS analysis by using reversed phase mobile phase solvents, and the chromatographic separation of the *cis/trans* isomers by using a chiral column (*i.e*., CHIRALPAK IB N-3). Although chiral columns are generally used for enantiomer separation^26–28^, chiral columns can also be used to separate diastereomers and achiral molecules, as shown by our studies and others^10, 11, 29^. In our previous study, the *cis/trans* isomers of phospholipid hydroperoxides bearing 13-HPODE (*i.e*., 16:0/13-9*Z*,11*E*-HPODE phosphocholine and 16:0/13-9*E*,11*E*-HPODE phosphocholine) were separated by CHIRALPAK IB-3^11^. Thus, in this study, we used CHIRALPAK IB N-3, which is the improved column of CHIRALPAK IB-3, assuming that the separation of HPODE *cis/trans* isomers can be achieved. As expected, HPODE isomers were clearly separated by CSP-LC-MS/MS (Fig. 2). From the results, it was suggested that the geometric isomerism of the carbon-to-carbon double bond in lipid hydroperoxides was discriminated and separated by cellulose tris (3,5-dimethylphenylcarbamate), which is the chiral selector of CHIRALPAK IB-3 and CHIRALPAK IB N-3. Thus, it can be assumed that these columns can achieve the separation of lipid hydroperoxide *cis/trans* isomers by interacting with fatty acids themselves or fatty acid molecules contained in lipid molecules. The detection limit of each HPODE isomer were in pmol levels. Since our newly established method enabled the analysis of HPODE isomers (Fig. 1), we then evaluated the lipid oxidation mechanisms in edible oils.

### Analysis and evaluation of lipid oxidation mechanisms in edible oils

Edible oils (soybean oil (SO), rice bran oil (RBO) and olive oil (OO)) were first subjected to light-exposure or heating, and were treated with Lipase AY-30. Lipase AY-30 hydrolyzes the ester bonds in triacylglycerol regardless of the fatty acid position or chain length^30^. Therefore, it can be assumed that all triacylglycerols were hydrolyzed into a crude sample containing glycerol and fatty acids including HPODE isomers. The progress of the hydrolysis reaction was confirmed by using photo-oxidized trilinolein as a model of oxidized triacylglycerol (data not shown). To the best of our knowledge, this is the first study applying lipase to analyze HPODE isomers from oxidized edible oils. The use of lipase may also be useful for the analysis of other complex lipid hydroperoxides (*e.g*., phospholipid hydroperoxides, triacylglycerol hydroperoxides and cholesterol hydroperoxides).

**Figure 3:**
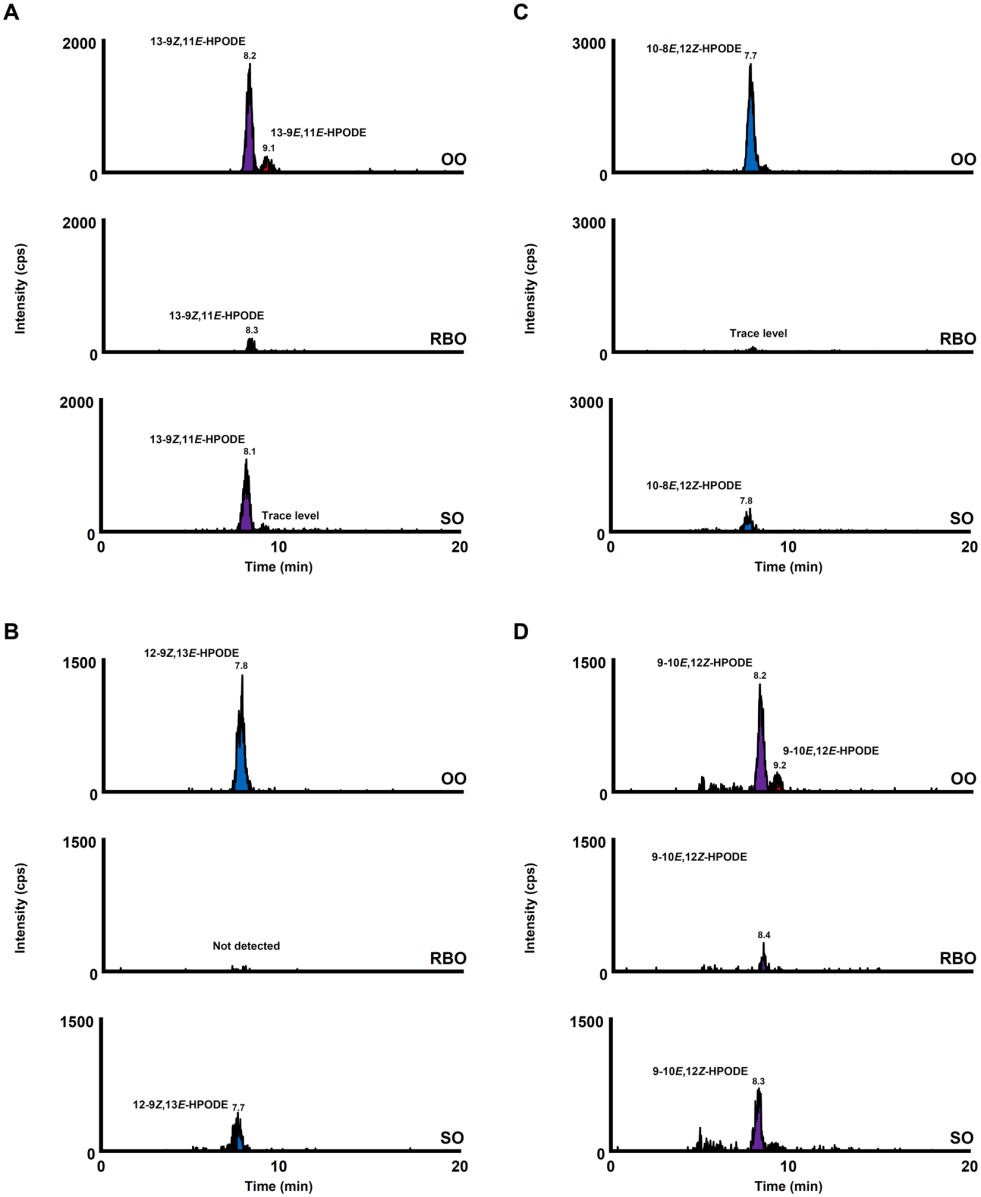
CSP-LC-MS/MS analysis of HPODE isomers in unoxidized edible oils. HPODE isomers were determined using the SRM mode as follows: 13-9*Z*,11*E*-HPODE and 13-9*E*,11*E*-HPODE, *m/z* 335.2 > 247.1 (**A**); 12-9*Z*,13*E*-HPODE, *m/z* 335.2 > 206.1 (**B**); 10-8*E*,12*Z*-HPODE, *m/z* 335.2 > 207.1 (**C**); 9-10*E*,12*Z*-HPODE and 9-10*E*,12*E*-HPODE, *m/z* 335.2 > 195.1 (**D**). Detailed analytical conditions are described in the Materials and Method section.

**Figure 4:**
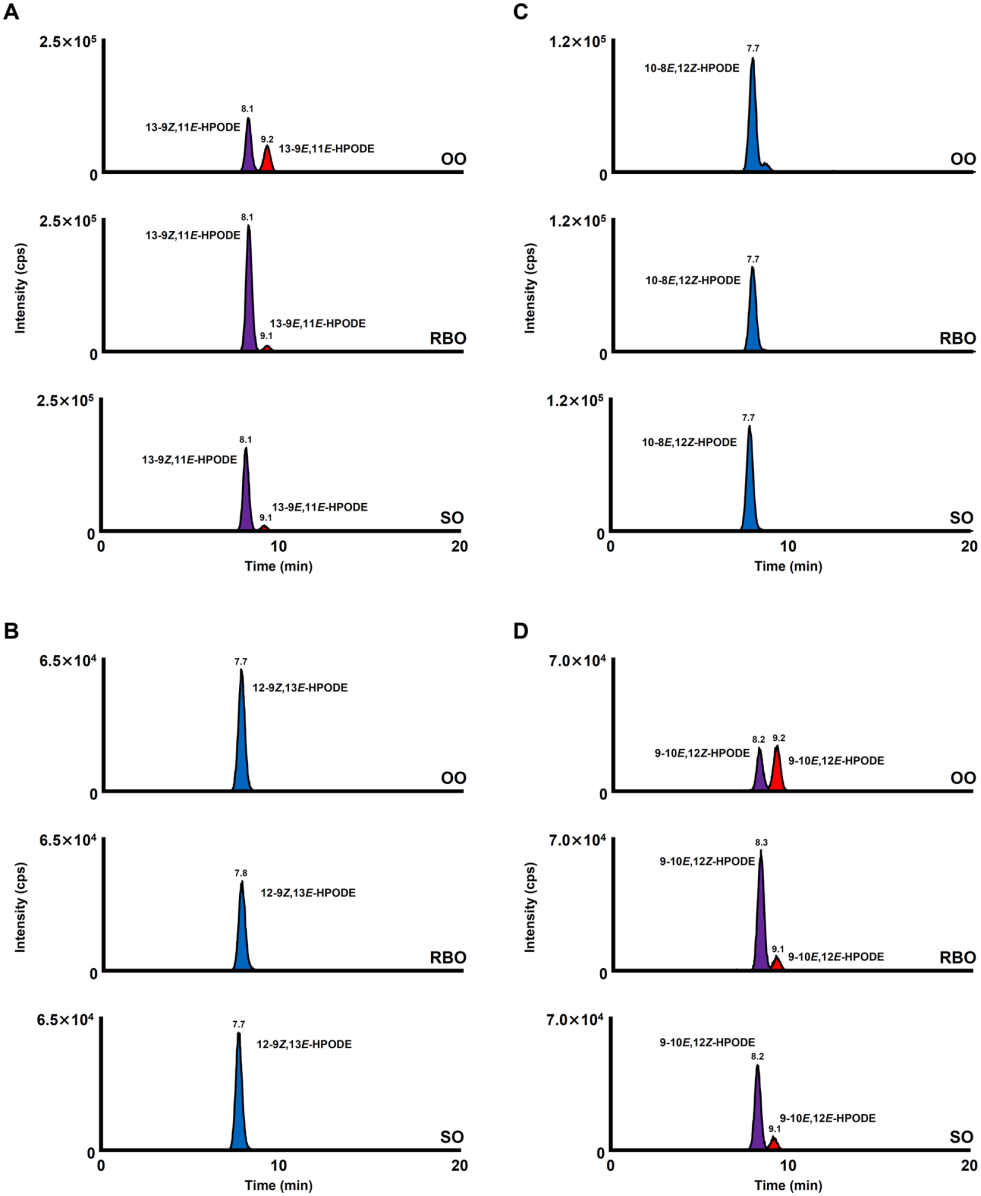
CSP-LC-MS/MS analysis of HPODE isomers in light-exposed edible oils. HPODE isomers were determined using the SRM mode as follows: 13-9*Z*,11*E*-HPODE and 13-9*E*,11*E*-HPODE, *m/z* 335.2 > 247.1 (**A**); 12-9*Z*,13*E*-HPODE, *m/z* 335.2 > 206.1 (**B**); 10-8*E*,12*Z*-HPODE, *m/z* 335.2 > 207.1 (**C**); 9-10*E*,12*Z*-HPODE and 9-10*E*,12*E*-HPODE, *m/z* 335.2 > 195.1 (**D**). Detailed analytical conditions are described in the Materials and Method section.

**Figure 5:**
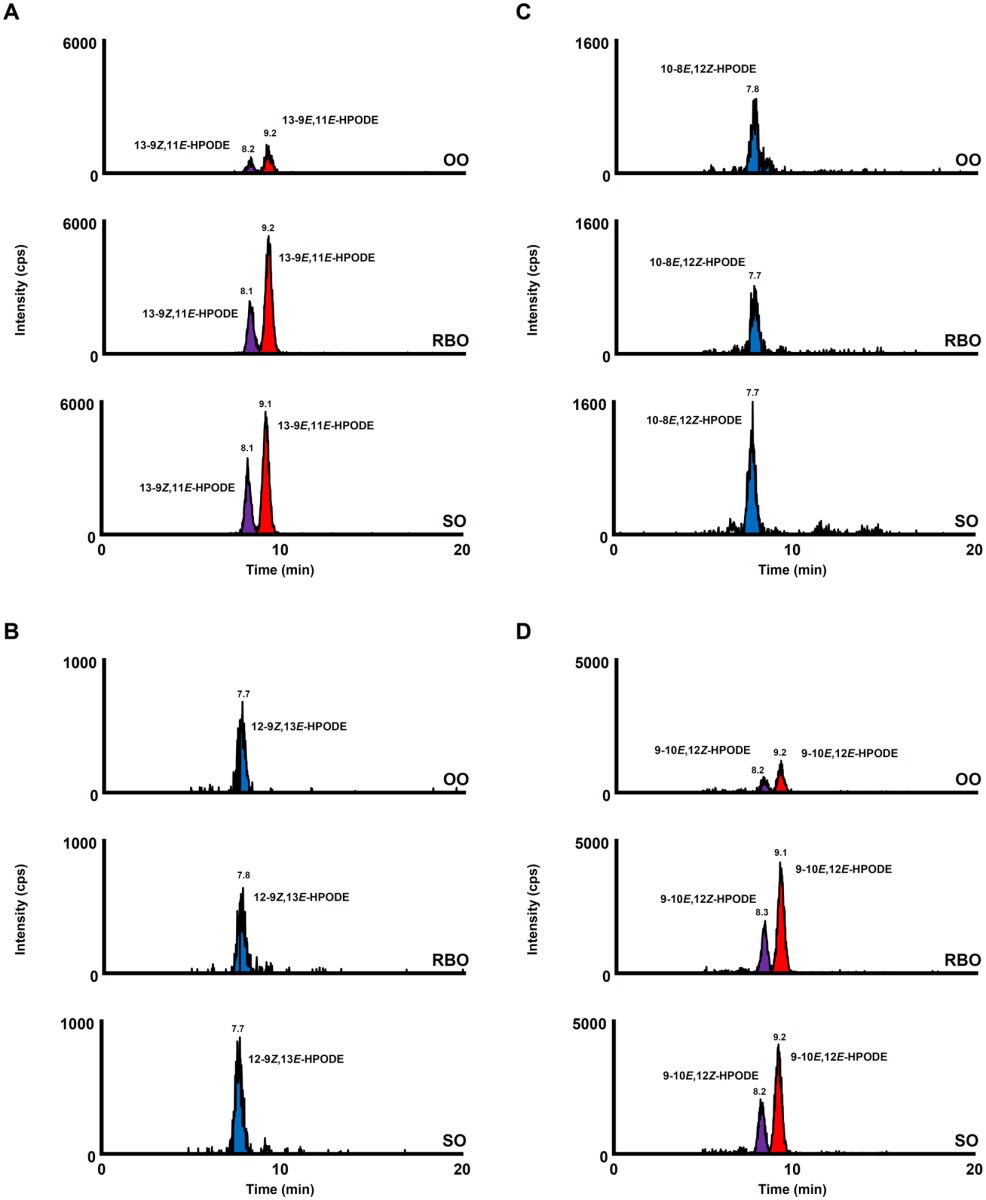
CSP-LC-MS/MS analysis of HPODE isomers in heated edible oils. HPODE isomers were determined using the SRM mode as follows: 13-9*Z*,11*E*-HPODE and 13-9*E*,11*E*-HPODE, *m/z* 335.2 > 247.1 (**A**); 12-9*Z*,13*E*-HPODE, *m/z* 335.2 > 206.1 (**B**); 10-8*E*,12*Z*-HPODE, *m/z* 335.2 > 207.1 (**C**); 9-10*E*,12*Z*-HPODE and 9-10*E*,12*E*-HPODE, *m/z* 335.2 > 195.1 (**D**). Detailed analytical conditions are described in the Materials and Method section.

HPODE isomers were detected in SO and OO (Fig. 3), despite being analyzed immediately after opening. The HPODE isomers that were mainly detected in unoxidized oils were the isomers characteristic to photo-oxidation (*e.g*., 13-9*Z*,11*E*-HPODE, 12-9*Z*,13*E*-HPODE, 10-8*E*,12*Z*-HPODE and 9-10*E*,12*Z*-HPODE). This implies that photo-oxidation had already occurred in edible oils before opening (*e.g*., during manufacturing process, transportation and/or sales). In light-exposed edible oils, these isomers specific to photo-oxidation were found in larger amounts compared to unoxidized oils (Fig. 4, Supplementary Information 2). Interestingly, light-exposed edible oils contained small amounts of 13-9*E*,11*E*-HPODE and 9-10*E*,12*E*-HPODE, which are isomers that result from the auto-oxidation of LA (Fig. 4, Supplementary Information 2). This suggests that the lipid hydroperoxides generated by photo-oxidation further became lipid peroxyl radicals, which are free radicals that can initiate the auto-oxidation (radical oxidation) of other lipids^1^. These results imply that light illumination (*e.g*., during sales and putting in the kitchen) could induce photo-oxidation as well as a small degree of auto-oxidation of edible oils. On the other hand, in edible oils subjected to heating, the HPODE isomers characteristic to auto-oxidation (*i.e*., 13-9*E*,11*E*-HPODE and 9-10*E*,12*E*-HPODE) were largely increased (Fig. 5, Supplementary Information 2). The HPODE isomers specific to photo-oxidation (*i.e*., 12-9*Z*,13*E*-HPODE and 10-8*E*,12*Z*-HPODE) were also detected in heated edible oils, but considering their low contents (Supplementary Information 2), these isomers were presumably the isomers that were present before the oils were heated (*i.e*., present before opening). These results indicate that the heating of edible oils (*e.g*., during cooking) could induce the auto-oxidation of edible oils.

With regard to vitamin E homologue levels, SO and RBO contained about 100 mg/100 g of vitamin E homologues. Vitamin E homologues are famous antioxidants in foods, particularly effective to auto-oxidation^31, 32^. One characteristic of RBO is the fact that RBO contains not only tocopherols but also tocotrienols. Tocotrienols are known to demonstrate similar antioxidative activities as tocopherols^31, 32^, and therefore the amounts of HPODE isomers characteristic to auto-oxidation (*i.e*., 13-9*E*,11*E*-HPODE and 9-10*E*, 12*E*-HPODE) did not differ with SO (Supplementary Information 2). On the other hand, OO contained lower levels of vitamin E homologues compared with SO and RBO (Supplementary Information 3). This was reflected in the ratio of 13-9*Z*,11*E*-HPODE to 13-9*E*,11*E*-HPODE and 9-10*E*,12*Z*-HPODE to 9-10*E*,12*E*-HPODE, of which the ratios were distinctly different between light-exposed SO and OO (Figs 4 and 5, Supplementary Information 2). Because 13-9*E*,11*E*-HPODE and 9-10*E*,12*E*-HPODE are isomers characteristic to auto-oxidation, high contents of these isomers indicate that auto-oxidation occurred at higher rates in light-exposed OO compared to SO and RBO. These results suggest that the auto-oxidation of edible oils are affected by the amounts of vitamin E homologues rather than the composition of vitamin E homologues.

Also, it is worth noting that HPODE isomers were detected in each OO sample despite containing only 6% of LA. This content of LA was significantly lower than that of SO and RBO (53% and 35% respectively) (Supplementary Information 4). However, the fact that OO contained similar HPODE amounts compared to SO and RBO indicates that OO was more susceptible to lipid oxidation than SO and RBO (Supplementary Information 2). One cause of this lipid oxidation may be the natural components in OO, which may act as photosensitizers that induce photo-oxidation^33^. These results suggest, for the first time, that the oxidation mechanisms of lipids (*i.e*., edible oils) can be evaluated by analyzing the characteristic positional and *cis/trans* isomers of lipid hydroperoxides (*i.e*., HPODE isomers). Thereby, the combination of the CSP-LC-MS/MS method and lipase may be useful for the evaluation of lipid oxidation mechanisms of foods.

## Conclusions

In this study, we developed the CSP-LC-MS/MS method to analyze the positional and *cis/trans* isomers of HPODE with the use of a chiral column and alkali metals (*i.e*., sodium ion). The combination of the CSP-LC-MS/MS with lipase enabled the understanding of lipid oxidation mechanisms (*i.e*., photo- and auto- oxidation) in oxidized edible oils. Thus, the herein developed method is a powerful and novel tool to evaluate the involvement of lipid oxidation in food deterioration and may be further applied to evaluate biological systems.

## Materials and Method

### Reagents

LA was obtained from Sigma-Aldrich (St. Louis, MO, USA). Rose bengal was purchased from Wako (Osaka, Japan). Lipase AY-30 was obtained from Amano Enzyme Inc. (Aichi, Japan). All other reagents were of the highest grade available.

### Preparation of HPODE isomer standards

HPODE isomers (Fig. 1) were prepared according to a previous method with slight modifications^18^. In brief, LA (10 g) was dissolved in 11 mL of methanol containing 0.9 mM rose bengal. The sample was photo-oxidized with 15,000 lux illumination intensity for 24 h at 4 °C. After photo-oxidation, 110 mL of hexane and 110 mL of water were added to extract oxidized LA including HPODE isomers. The sample was vortexed and partitioned by centrifugation (1,000 × *g*, 10 min, 4 °C) into two layers: the hexane layer (upper layer) and the methanol–water layer (lower layer). The upper hexane layer (lipid fraction) was collected and evaporated. The resultant sample (7.4 g) was dissolved in methanol, loaded onto a methanol-equilibrated Sep-Pak QMA cartridge (360 mg, Waters, Milford, MA) and eluted with 5 mL of methanol. Rose bengal was retained on the cartridge, while the eluate containing HPODE was collected. The eluate was evaporated and dissolved in 33 mL of hexane–2-propanol (100:1, v/v). To isolate the HPODE isomers, the sample was injected into a semipreparative LC system, which included a LC-6AD pump (Shimadzu, Kyoto, Japan) and a SPD-20A UV detector (Shimadzu). Two columns (Silica SG120 [5 µm, 10 × 250 mm, Shiseido, Tokyo, Japan] and Inertsil SIL-100A [5 µm, 10 × 250 mm, GL Sciences Inc., Tokyo, Japan]) were connected and used at 40 °C to obtain good separation. The mobile phase was hexane–2-propanol–acetic acid (100:1:0.1, v/v/v) eluted at 18 mL/min. HPODE isomers were detected by the UV absorbance at 210 nm. Because 9-10*E*,12*Z*-HPODE and 9-10*E*,12*E*-HPODE were not completely separated by silica columns, 9-10*E*,12*Z*-HPODE and 9-10*E*,12*E*-HPODE were collected together as one fraction, evaporated, dissolved in methanol and subjected to semipreparative HPLC under the following conditions: column, C18 column (Wakosil-II 5C18 RS-prep [5 µm, 20 mm × 250 mm, Wako, Osaka, Japan]); column temperature, 40 °C; mobile phase, methanol–water (10:3, v/v) containing 5 mM ammonium acetate; flow rate, 20 mL/min. The purity and chemical structure of each HPODE isomer was confirmed by MS/MS analysis using a micrOTOF-Q II mass spectrometer (Bruker Daltonik, Bremen, Germany) and by ^1^H NMR using a Varian Unity Plus-400 spectrometer (Varian, Palo Alto, CA, USA) at 400 MHz with chloroform-d_4_ (CDCl_3_) as the solvent. The prepared HPODE isomers were dissolved in methanol (1 mM each) and a portion (1 μL) of the standard solution was analyzed by CSP-LC-MS/MS.

### CSP-LC-MS/MS analysis of HPODE isomers

A Shimadzu liquid chromatography system (Shimadzu, Kyoto, Japan), consisting of a vacuum degasser, a quaternary pump and an autosampler, was connected to a micrOTOF-Q II mass spectrometer. HPODE isomers were analyzed using a chiral column (CHIRALPAK IB N-3 [3 µm, 2.1 × 150 mm, Daicel, Osaka, Japan]) eluted with methanol–water–formic acid (82:18:0.1, v/v/v) at a flow rate of 0.2 mL/min. The column temperature was maintained at 40 °C. The column eluent was mixed with a post-column reagent containing sodium ions (*i.e*., methanol containing 2 mM sodium acetate) to enhance the fragmentation of HPODE isomers. The flow rate of the post-column reagent was 0.01 mL/min. HPODE isomers were detected using the SRM mode as follows: 13-9*Z*,11*E*-HPODE and 13-9*E*,11*E*-HPODE, *m/z* 335.2 > 247.1; 12-9*Z*,13*E*-HPODE, *m/z* 335.2 > 206.1; 10-8*E*,12*Z*-HPODE, *m/z* 335.2 > 207.1; 9-10*E*,12*Z*-HPODE and 9-10*E*,12*E*-HPODE, *m/z* 335.2 > 195.1. The MS/MS parameters used in the current study are shown in Table 1.

**Table 1:** Analytical conditions of HPODE isomers used for MS/MS analysis.

Parameters	Condition
Source	ESI
Ion polarity	Positive
Mass range (*m/z*)	50–500
End plate offset (v)	500
Capillary (v)	4500
Nebulizer^16^	1.6
Dry gas (L/min)	8.0
Dry temp (°C)	180
Funnel 1RF (Vpp)	200.0
Funnel 2RF (Vpp)	200.0
Hexapole RF (Vpp)	100.0
isCID energy (eV)	0.0
Ion energy (eV)	2.0
Low mass (*m/z*)	50.00
Collision energy (eV)	10.0
Collision RF (Vpp)	280.0
Transfer time (μs)	31.0
Pre pulse storage (μs)	9.5
Width	1.0
Collision	14.0

ESI, electrospray ionization; RF, radio frequency; isCID, in-source collision induced dissociation.

### Analysis of oxidized edible oils by CSP-LC-MS/MS

RBO and OO were purchased from local markets in Sendai, Japan. SO was obtained from Wako (Osaka, Japan). The fatty acid composition of the edible oils was analyzed by previously reported methods^34^. The vitamin E homologue (*i.e*., tocopherols and tocotrienols) contents of the edible oils was analyzed by LC-MS/MS using a 4000 QTRAP quadrupole/linear ion-trap tandem mass spectrometer (AB SCIEX, Tokyo, Japan). Approximately 100 mg of edible oil was weighed and dissolved in 2 mL of n-hexane. The solution was further 50-fold diluted with n-hexane. A portion (10 μL) of the prepared solution was subjected to LC-MS/MS. Separation was performed at 40 °C using a silica column (Inertsil SIL-100A [5 µm, 4.6 × 250 mm; GL Sciences Inc., Tokyo, Japan]). A mixture of hexane–1,4-dioxane–2-propanol (100:4:0.5, v/v/v) was used as the mobile phase at a flow rate of 1.0 mL/min. Vitamin E homologue were detected with atmospheric pressure chemical ionization (APCI), operated in the negative ion mode. The MS/MS parameters and SRM pairs for vitamin E homologue are shown in Supplementary Information 5. Oxidized edible oils were prepared as follows: 5 g of edible oil was placed on a petri dish and was subjected to either light-exposure (under a light irradiated condition (10,000 lux) at 25 °C for 4 days) or heating (under a light-shielded condition at 180 °C for 30 min). Unoxidized edible oils (collected immediately after opening) and oxidized edible oils (400 μg each) were dissolved in 100 μL of hexane containing 0.002% butylated hydroxytoluene. Lipase AY-30 (200 mg) dissolved in 1 mL of 50 mM phosphate buffer (pH 6.8) was added to the solution and vortexed at 25 °C for 10 min. Subsequently, 1,500 μL of diethyl ether–acetic acid (99:1, v/v) was added to sample, vortexed and partitioned by centrifugation (1,940 × *g*, 5 min, 4 °C). After centrifugation, the upper diethyl ether layer (fatty acid and HPODE fraction) was collected. The remaining aqueous layer was re-extracted with diethyl ether (1,500 μL) and subjected to centrifugation. The combined upper layer fraction was evaporated and dissolved in 100 μL of methanol, and analyzed by CSP-LC-MS/MS as described above.

## Electronic supplementary material

Supplementary information
