# Supplementary material for: A novel chiral stationary phase LC-MS/MS method to evaluate oxidation mechanisms of edible oils
Source: Sci Rep. 2017 Aug 30;7:10026. doi: 10.1038/s41598-017-10536-2 (PMC5577281; doi:10.1038/s41598-017-10536-2)
Supplement: Supplementary file 1 — Supplementary information [file 41598_2017_10536_MOESM1_ESM.pdf]

*Scientific Reports (Research Paper)*

# A novel chiral stationary phase LC-MS/MS method to evaluate oxidation mechanisms of edible oils.

Junya Ito [a], Naoki Shimizu [a], Eri Kobayashi [a], Yasuhiko Hanzawa [a], Yurika Otoki [a], Shunji Kato [a,b], Takafumi Hirokawa [c], Shigefumi Kuwahara [c], Teruo Miyazawa [a,d], Kiyotaka Nakagawa [a,*]

[a] *Food and Biodynamic Chemistry Laboratory, Graduate School of Agricultural Science, Tohoku University, Sendai, 980-0845, Japan*

[b] *Department of Cell Biology, Division of Host Defense Mechanism, Tokai University School of Medicine, Isehara, Kanagawa, 259-1193, Japan*

[c] *Laboratory of Applied Bioorganic Chemistry, Graduate School of Agricultural Science, Tohoku University, Sendai, 980-0845, Japan*

[d] *New Industry Creation Hatchery Center (NICHe), Tohoku University, Sendai, 980-8579, Japan*

*Corresponding Author

Kiyotaka Nakagawa, Ph.D.

Professor

Food and Biodynamic Chemistry Laboratory,

Graduate School of Agricultural Science, Tohoku University

468-1 Aramaki Aza Aoba, Aoba-Ku, Sendai 980-0845, Japan

Phone: +81-22-757-4416 Fax: +81-22-757-4417

E-mail: nkgw@m.tohoku.ac.jp

**Supplementary Information 1**

## α-cleavage

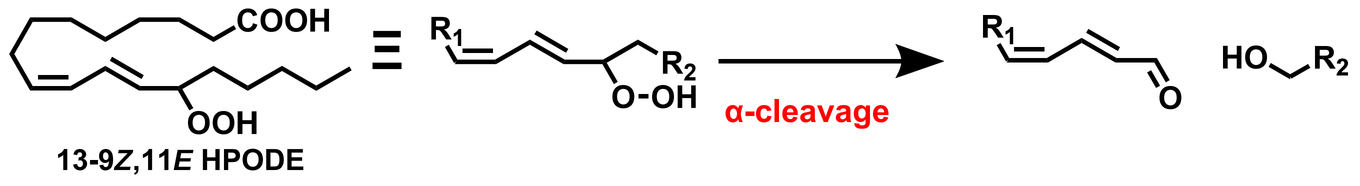


## Hock cleavage

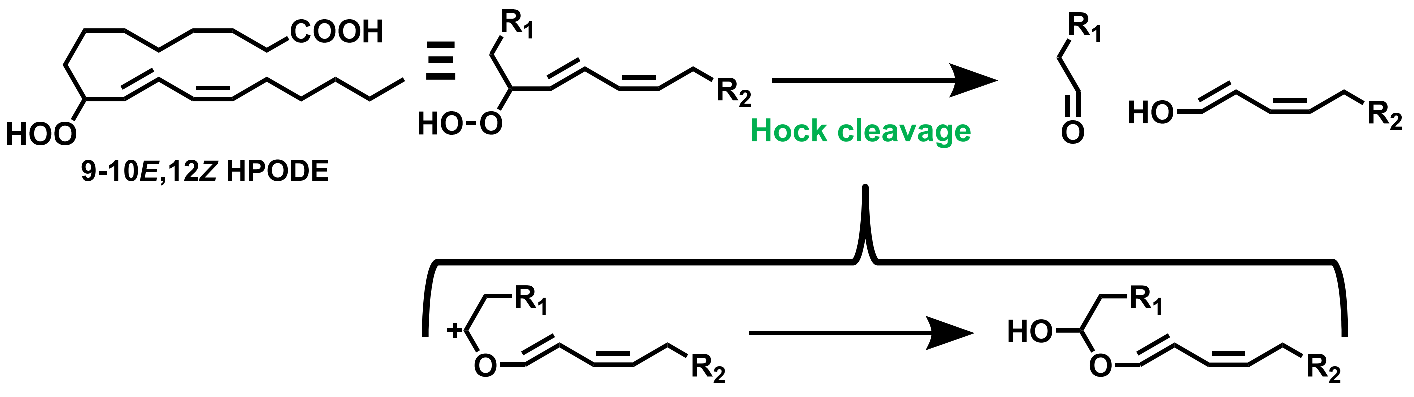


The estimated fragmentation mechanisms during MS/MS analysis of HPODE isomers in the presence of sodium ion.

**Supplementary Information 2. Peak areas of HPODE isomers in edible oils.**

| | | Auto-oxidation products | | Auto- and Photo-oxidation products | | Photoo-oxidation products | |
|---|---|---|---|---|---|---|---|
| | | 13-9E,11E-HPODE | 9-10E,12E-HPODE | 13-9Z,11E-HPODE | 9-10E,12Z-HPODE | 12-9Z,13E-HPODE | 10-8E,12Z-HPODE |
| | | | | Peak area | | | |
| SO | Unoxidized | 1.4E+03 | 2.1E+03 | 2.2E+04 | 1.5E+04 | 7.8E+03 | 9.4E+03 |
| | Light-exposed | 2.6E+05 | 1.7E+05 | 3.6E+06 | 1.1E+06 | 1.5E+06 | 2.5E+06 |
| | Heated | 1.3E+05 | 1.0E+05 | 6.9E+04 | 4.7E+04 | 2.2E+04 | 3.5E+04 |
| RBO | Unoxidized | not detected | not detected | 3.2E+03 | 3.1E+03 | not detected | 1.3E+03 |
| | Light-exposed | 2.9E+05 | 2.0E+05 | 5.5E+06 | 1.6E+06 | 1.2E+06 | 2.0E+06 |
| | Heated | 1.3E+05 | 1.0E+05 | 5.5E+04 | 4.2E+04 | 1.5E+04 | 2.1E+04 |
| OO | Unoxidized | 5.1E+03 | 4.2E+03 | 3.2E+04 | 2.4E+04 | 2.7E+04 | 5.4E+04 |
| | Light-exposed | 1.3E+06 | 6.6E+05 | 2.3E+06 | 5.9E+05 | 1.6E+06 | 3.0E+06 |
| | Heated | 2.7E+04 | 2.4E+04 | 1.3E+04 | 1.3E+04 | 1.4E+04 | 2.5E+04 |

**Supplementary Information 3. Vitamin E homologue contents of the edible oils.**

| | SO | RBO | OO |
|---|---|---|---|
| | | (mg/100 g oil) | |
| α-Toc | 14.0 ± 0.5 | 47.3 ± 1.9 | 7.3 ± 0.2 |
| β-Toc | 0.4 ± 0.1 | 0.5 ± 0.1 | n.d. |
| γ-Toc | 71.3 ± 3.0 | 6.8 ± 0.2 | 2.4 ± 0.0 |
| δ-Toc | 14.4 ± 1.0 | n.d. | n.d. |
| α-T3 | n.d. | 19.3 ± 0.6 | n.d. |
| β-T3 | n.d. | n.d. | n.d. |
| γ-T3 | n.d. | 27.2 ± 2.6 | n.d. |
| δ-T3 | n.d. | 0.3 ± 0.1 | n.d. |
| Total | 99.9 ± 4.4 | 101.4 ± 4.5 | 9.8 ± 0.2 |

Means ± SD (n = 3). Toc, tocopherol; T3, tocotrienol; n.d., not detected.

**Supplementary Information 4. Fatty acid composition of the edible oils.**

| | SO | RBO | OO |
|---|---|---|---|
| | | (%) | |
| C16:0 | 10.5 ± 0.0 | 16.3 ± 0.0 | 12.1 ± 0.0 |
| C16:1 n-7 | n.d. | n.d. | 1.0 ± 0.0 |
| C18:0 | 3.7 ± 0.0 | 1.9 ± 0.0 | 3.2 ± 0.0 |
| C18:1 n-9 | 23.0 ± 0.1 | 42.4 ± 0.0 | 73.8 ± 0.1 |
| C18:1 n-7 | 1.5 ± 0.0 | 0.9 ± 0.0 | 2.3 ± 0.1 |
| C18:2 n-6 | 53.0 ± 0.1 | 35.0 ± 0.0 | 6.3 ± 0.0 |
| C18:3 n-3 | 6.5 ± 0.0 | 1.2 ± 0.0 | n.d. |
| C20:0 | n.d. | 0.7 ± 0.0 | n.d. |
| C20:1 n-9 | n.d. | 0.6 ± 0.0 | n.d. |
| Others | 1.8 ± 0.1 | 1.1 ± 0.0 | 1.3 ± 0.0 |

Means ± SD (n = 3). n.d., not detected.

**Supplementary Information 5. Analytical conditions of vitamin E homologue used for MS/MS analysis**

| | α-Toc | β-Toc | γ-Toc | δ-Toc | α-T3 | β-T3 | γ-T3 | δ-T3 |
|---|---|---|---|---|---|---|---|---|
| Precursor ion (*m/z*) | 429.3 | 415.3 | 415.3 | 401.3 | 423.3 | 409.2 | 409.2 | 395.2 |
| Production (*m/z*) | 163.0 | 149.1 | 148.9 | 135.0 | 163.1 | 148.8 | 148.9 | 134.7 |
| Declustering potential (V) | -80 | -80 | -80 | -85 | -80 | -90 | -85 | -80 |
| Entrance potential (V) | -10 | -10 | -10 | -10 | -10 | -10 | -10 | -10 |
| Collision energy (V) | -38 | -38 | -42 | -40 | -38 | -34 | -40 | -36 |
| Collision cell exit potential (V) | -7 | -5 | -5 | -3 | -1 | -5 | -5 | -5 |
| Curtain gas (psi) | 30 | | | | | | | |
| Collision gas (psi) | 3 | | | | | | | |
| Ion spray voltage (V) | -4500 | | | | | | | |
| Temperature (°C) | 400 | | | | | | | |
| Ion source gas 1 (psi) | 60 | | | | | | | |
| Ion source gas 2 (psi) | 40 | | | | | | | |

Toc, tocopherol; T3, tocotrienol
